# Surgery in Battle Areas

**Published:** 1918-10

**Authors:** 


### WAR MEDICINE

Published by the American Red Cross Society in France
for the
Medical Officers of the American Expeditionary Forces

EDITORIAL OFFICES: 2, Place de Rivoli, Paris, Rooms 422-425

WAR MEDICINE *accepts no original articles. Its scope is limited to the publication of the proceedings of the Research Society of the American Red Cross in France, abstracts of original articles, and editorial comment on subjects pertaining to the medicine, surgery, and hygiene of the war.*

*Circulars, bulletins, and reports from the Office of the Chief Surgeon of the American Expeditionary Forces will also appear in* WAR MEDICINE.

WAR MEDICINE *is distributed free of charge to the medical officers of the American Expeditionary Forces.*

*All communications pertaining to the mailing list should be addressed to the Editorial Offices. If copies are not received promptly and regularly the managing editor should be informed at once. Addresses should be written distinctly.*

THE RESEARCH SOCIETY OF THE AMERICAN RED CROSS IN FRANCE

*All communications should be addressed to the Secretary,*
2, *Place de Rivoli, Paris, Room* 422

### EDITORIAL COMMENTS

### SURGERY IN BATTLE AREAS

"The Creed for Medical Officers of the American Expeditionary Forces, operating at the front, has been written", said a prominent American surgeon, after the Conference on Surgery in Battle Areas at the September meeting of the Research Society. This opinion was concurred in generally by others who had the privilege of hearing the prompt and decisive answers to various surgical problems that were discussed by the French, British, and American surgeons who took part in the Conference.

This surgical "Creed", like those for sects or religions, was not all written in a day, historic as the occasion of this Conference may have been. It is not considered perfect and is subject to revision as advances in surgery are made, but it represents the sum of the applied knowledge regarding surgery in battle areas from the time when the priests cared for wounded soldiers of the hosts of Rameses I to the era of the highly trained and technical surgeons who are today directing the surgical services in the Armies of the Allies under Marshal Foch. Nor was it written by one man, or a group of men. Pasteur and Lister, more than any living men, had to do with its making; and other celebrated scientists, renowned clinicians and great military surgeons, whose names are a part of the history of medicine and surgery, also took part in preparing this " Creed " for surgeons in battle areas. Neither were there present at the Conference all those who have had the largest experience in dealing with war wounds; because the great surgeons from the Allied Armies, who announced the answers to the problems propounded, expressed the opinions of the surgical services which they represented; and their conclusions were arrived at only after years of experimental research by many laboratory workers, and clinical experience by a large number of surgeons in dealing with hundreds of thousands of wounded men.

### A Carefully Prepared Surgical Creed

The questions relating to surgery in battle areas that were asked at the Conference were not hastily prepared. Groups of men from various hospitals, and representing several subcommittees of the Research Committee that are working on surgical problems, had been discussing plans for this Conference for months before it was called.

Weeks before the meeting of the Research Society typewritten copies of the questions relating to surgical problems in battle areas, that were printed on the program of the Conference, were sent to a number of surgeons in the French, British, and American Armies. Replies had been received from some of the ablest surgeons in the three armies before the meeting, and the representatives who took part in the Conference were prepared to give the answers for their respective Armies.

Following the meeting of the Research Society, a committee, after having carefully considered the stenographic reports of the discussions at the Conference, and having read replies that had been received before the meeting, prepared the answers to the problems. The questions and the answers were then carried to Professional Headquarters where a conference was held with those in authority there, and the "Creed" was made to fit conditions in the American Expeditionary Forces. It is therefore evident that no document could carry greater weight of authority, and that, in the report of this Conference, the surgeons of the American Expeditionary Forces have a code of action by which to live in performing their duties in battle areas.

### For Surgeons in Battle Areas.

Having a "Creed" that has been prepared with such care, it is clearly the duty of medical officers to learn it and to follow its teachings; as completely as any religious enthusiast ever lived up to the mandates of the leaders of his faith. Each and every surgeon in the American Expeditionary Forces should not only read the report of the "Conference on Surgery in Battle Areas", but should also study it until he knows the problems and their answers so thoroughly that in times of stress of battle nothing, not even the bursting of bombs from the German Gothas around the hospital or clearing station where he is working, could make him deviate from its teaching. It should be as natural for him to carry out this surgical "Creed" as it is to disinfect his hands before performing an abdominal operation.

Reprints from *War Medicine* containing the report of this "Conference on Surgery in Battle Areas" will be sent to every surgeon who has to deal with war wounds. In the relatively quiet periods at the front, division surgeons and commanding officers of hospitals might do well to have medical officers under them, who have any part whatever in dealing with wounded men, study this report, and require them to stand oral or written examinations on the problems discussed in it.

This "Creed" for surgeons operating at the front has been written, is not regarded as perfect by those who have had a part in its making; and it is not intended to interfere with the individualism of the surgeon; because every case, after all, presents a problem which must be dealt with according to the judgment of the surgeon, whose conscience should direct him to do with the wounded patriot as he "would have done unto him", if he could put himself in the soldier's place.

### A Tried Out Creed but Subject to Revision

Those who have prepared the questions and their answers relating to surgery in battle areas realize that there is much to learn regarding the treatment of war wounds, and every surgeon should feel it is his duty to make the effort to improve upon the methods advised in this report. Research workers in all the Allied armies will continue to direct their energies to surgical problems, and clinicians will be unceasing in their efforts to advance the practice of surgery, and as surgical technic and administrative methods are perfected this "Creed" will be revised.

There is no time, nor is it right to experiment with or to carry out new and individual ideas in treating wounded men in the push of battle. Only plans and methods which have been tried, and which have stood the test of many battles, with many workers, should be followed by surgeons in battle areas. This "Creed" expresses the faith of the masters in surgery at this time, and if its teachings are lived up to by the surgeons who have the responsibility of saving the limbs and lives of the brave men who are making the great sacrifice in the cause of liberty, many of the finest of America's manhood will be spared to fight, not only in war, but afterwards when men strong and true will be needed for the restoration and reconstruction of the homes, farms, and factories of the nations that have suffered to thwart the Prussian vandals in their efforts to conquer and overrun the world.

